# Neutrophils associate with Bowman’s capsule rupture specifically in PR3-ANCA glomerulonephritis

**DOI:** 10.1007/s40620-021-01208-6

**Published:** 2021-12-01

**Authors:** Samy Hakroush, Björn Tampe

**Affiliations:** 1 Institute of Pathology, University Medical Center Göttingen, Göttingen, Germany; 2 Department of Nephrology and Rheumatology, University Medical Center Göttingen, Göttingen, Germany

**Keywords:** ANCA glomerulonephritis, Bowman’s capsule rupture, Tubulointerstitial inflammation, Neutrophils

## Abstract

**Background:**

Renal involvement is a common and severe complication of ANCA (antineutrophil cytoplasmic antibody) associated vasculitis (AAV) potentially resulting in a pauci-immune necrotizing and crescentic antineutrophil cytoplasmic antibody (ANCA) glomerulonephritis (GN) with acute kidney injury (AKI), end-stage renal disease (ESRD) or death. We recently described that Bowman’s capsule rupture links glomerular damage to tubulointerstitial inflammation in ANCA-associated glomerulonephritis. Herein we provide a comprehensive histological subtyping of immune cell infiltrates in association with Bowman’s capsule rupture in ANCA GN.

**Methods:**

A total of 44 kidney biopsies with ANCA GN were retrospectively included in a single-center observational study. Within a renal biopsy specimen, each glomerulus was scored separately for the presence of extensive and focal Bowman’s capsule rupture in injured glomeruli. Infiltrates of neutrophils, eosinophils, plasma cells, and mononucleated cells (macrophages, lymphocytes) were quantified as a fraction of the area of total cortical inflammation.

**Results:**

Extensive Bowman’s capsule rupture was associated with tubulointerstitial inflammation containing infiltrates of neutrophils, eosinophils and plasma cells. A similar association was observed for the presence of focal Bowman’s capsule rupture, correlating with tubulointerstitial inflammation containing neutrophils, eosinophils and plasma cells. Multiple logistic regression confirmed that extensive Bowman’s capsule rupture correlated with tubulointerstitial inflammation containing neutrophils, and focal Bowman’s capsule rupture correlated with neutrophil and plasma cell infiltration. Furthermore, this association was specifically observed in PR3-ANCA GN.

**Conclusion:**

To our knowledge, this is the first report linking Bowman’s capsule rupture directly to tubulointerstitial inflammation by immune cell subtypes. This underscores a pathomechanistic link between tubulointerstitial and glomerular lesions in ANCA GN and needs further investigation.

**Graphical abstract:**

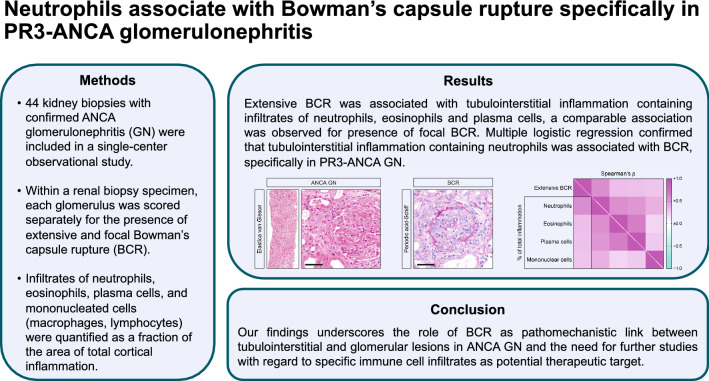

## Introduction

Antineutrophil cytoplasmic antibody (ANCA)-associated vasculitis (AAV) is a small vessel vasculitis according to the 2012 revised Chapel Hill Consensus Conference Nomenclature of Vasculitides, most frequently presenting as microscopic polyangiitis (MPA) or granulomatosis with polyangiitis (GPA) [1, 2]. Renal involvement is a common and severe complication of AAV potentially resulting in pauci-immune necrotizing and crescentic ANCA glomerulonephritis (GN) with acute kidney injury (AKI), end-stage renal disease (ESRD) or death [2]. Clinicopathologic studies of the European Vasculitis Study Group (EUVAS) demonstrated that distinct glomerular lesions are related to renal outcome in ANCA GN [3–6]. Derived from these studies, histopathological subgrouping into four classes (focal, crescentic, mixed, and sclerotic) as defined by Berden et al. was shown to predict long-term renal survival rates [7]. However, multivariable analyses demonstrated no improvement in outcome prediction in most of these studies, mainly attributed to no outcome difference in the crescentic and mixed classes [8–16]. Therefore, Brix et al. suggested the ANCA renal risk score (ARRS) by incorporating tubular atrophy/interstitial fibrosis (TA/IF) to the percentage of normal glomeruli and baseline glomerular filtration rate (GFR) to predict ESRD in patients with AAV, underscoring a pathomechanistic link between tubulointerstitial and glomerular lesions in ANCA GN [17]. Bowman’s capsule rupture was first described more than 30 years ago and we recently described that Bowman’s capsule rupture links glomerular damage to tubulointerstitial inflammation in ANCA-associated glomerulonephritis in a considerable subset of patients with ANCA GN [18]. An increased fraction of glomeruli affected by extensive Bowman’s capsule rupture in ANCA GN was associated with tubulointerstitial inflammation, suggesting that interstitial inflammation may also have predictive value in assessing the risk of decline in kidney function in ANCA GN [4, 10, 19]. Furthermore, we and others have previously described that focal Bowman’s capsule rupture with less extensive lesions was observed even more frequently in ANCA GN [20, 21]. The concept that tubulointerstitial injury mediates impairment of renal function was described more than five decades ago, in studies showing that decline of kidney function exhibited a stronger correlation with the severity of tubulointerstitial rather than with glomerular damage [22]. We recently characterized intrarenal subtypes of immune cell infiltrates in myeloperoxidase (MPO)-ANCA versus proteinase 3 (PR3)-ANCA GN, associated with distinct glomerular and tubulointerstitial lesions [23]. However, the association between Bowman’s capsule rupture and distinct immune cell subtypes has not been explored so far. Therefore, herein we provide comprehensive histological subtyping of immune cell infiltrates in association with extensive or focal Bowman’s capsule rupture in a cohort of 44 patients with ANCA GN confirmed by renal biopsy. The cohort comprises cases who underwent a kidney biopsy between 2015 and 2020 in a single-center, and this study represents a further step in the description of the relationship between Bowman’s capsule rupture and kidney damage [18].

## Methods

### Study population

A total of 44 kidney biopsies with confirmed renal involvement of ANCA GN at the University Medical Center Göttingen were retrospectively included between 2015 and 2020, the patient cohort has been previously described [18]. All studies involving human participants were reviewed and approved by the Institutional Review Board of the University Medical Center Göttingen, Germany (protocol numbers 22/2/14 and 28/09/17). Medical records were used to obtain data on age, sex, diagnosis (GPA or MPA) and laboratory results.

### Renal histopathology

A renal pathologist evaluated all biopsies and was blinded to all clinical data and analyses. Within a renal biopsy specimen, each glomerulus was scored separately for the presence of extensive and focal Bowman’s capsule rupture in injured glomeruli (crescentic and/or necrotic). Consequently, the percentage of glomeruli affected by Bowman’s capsule rupture was calculated as a fraction of the total number of glomeruli in each renal biopsy. Infiltrates of neutrophils, eosinophils, plasma cells, and mononucleated cells (macrophages, lymphocytes) were quantified as described previously [23]. The area of total cortical inflammation was quantified in each section stained with hematoxylin–eosin (HE) as a fraction of the total area. Distinct immune cell infiltrates were quantified as follows: neutrophils, eosinophils, plasma cells, and mononucleated cells (macrophages, lymphocytes) were identified by morphological criteria and fractions of the total cortical inflammation were estimated for each cell type.

### Statistical methods

Variables were tested for normal distribution using the Shapiro–Wilk test. Statistical comparisons were not formally powered or prespecified. Spearman correlation analyses were used to analyze correlations and shown by a heatmap reflecting mean values of Spearman’s ρ, asterisks indicate *p* < *0.05*. Data analyses were performed with GraphPad Prism (version 8.4.3 for MacOS, GraphPad Software, San Diego, California, USA). Multiple regression analyses were performed using IBM SPSS Statistics (version 27 for MacOS, IBM Corporation, Armonk, NY, USA). A probability (*p*) value of < 0.05 was considered statistically significant.

## Results

**Fig. 1:**
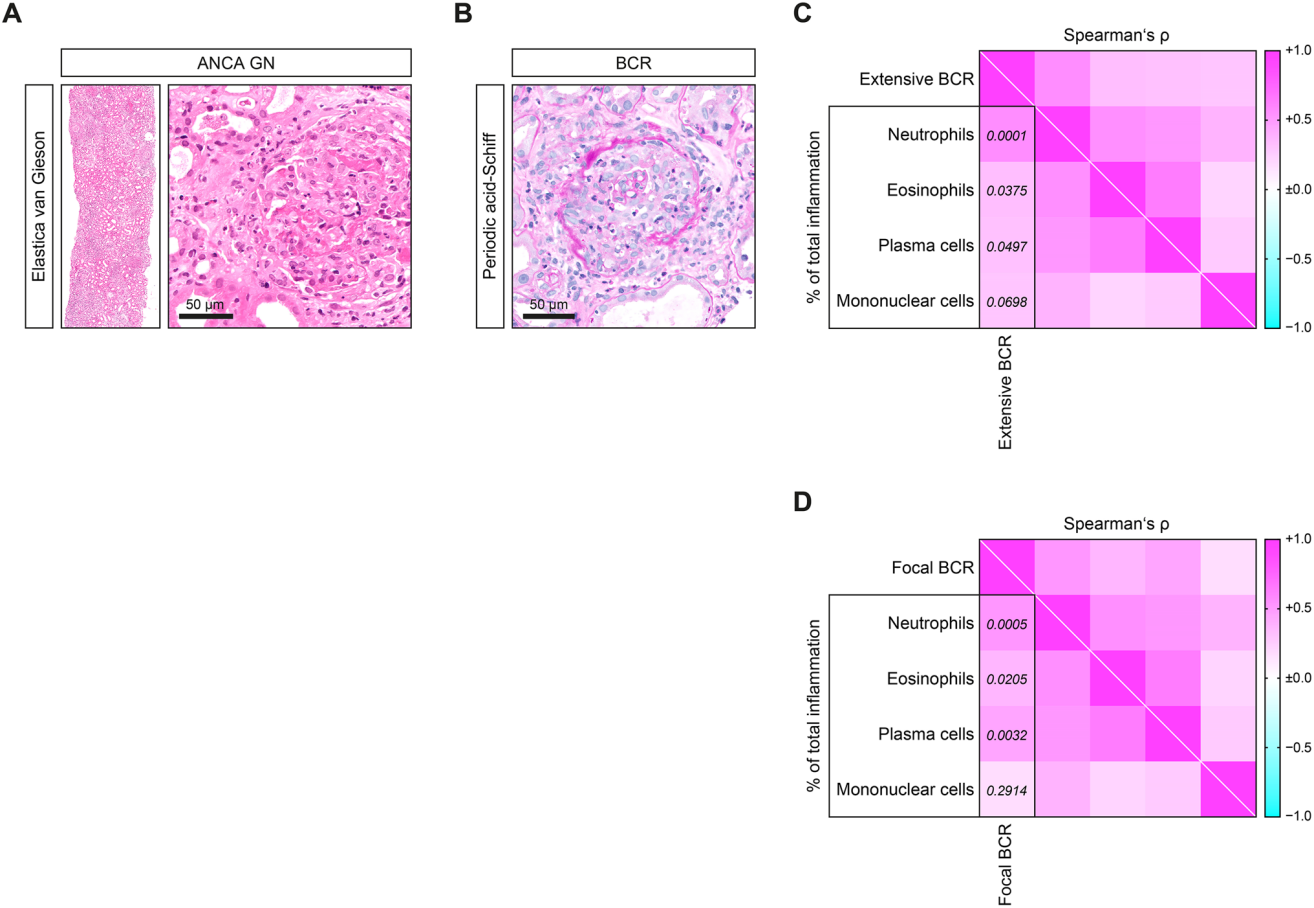
Tubulointerstitial inflammation containing neutrophils associates with BCR in ANCA GN. **A** Representative Elastica van Gieson-stained renal biopsy in ANCA GN (scale bar: 50 µm). **B** Representative periodic acid-Schiff reaction-stained renal biopsy in ANCA GN with BCR (scale bar: 50 µm). **C**, **D** The association between the fraction of glomeruli affected by extensive or focal BCR, and subtypes of immune cell infiltrates in ANCA GN is shown by a heatmap reflecting mean values of Spearman’s ρ, corresponding values of *p* are shown. *ANCA* anti-neutrophil cytoplasmic antibodies, *BCR* Bowman’s capsule rupture, *GN* glomerulonephritis, *MPO* myeloperoxidase, *PR3* proteinase 3

**Fig. 2:**
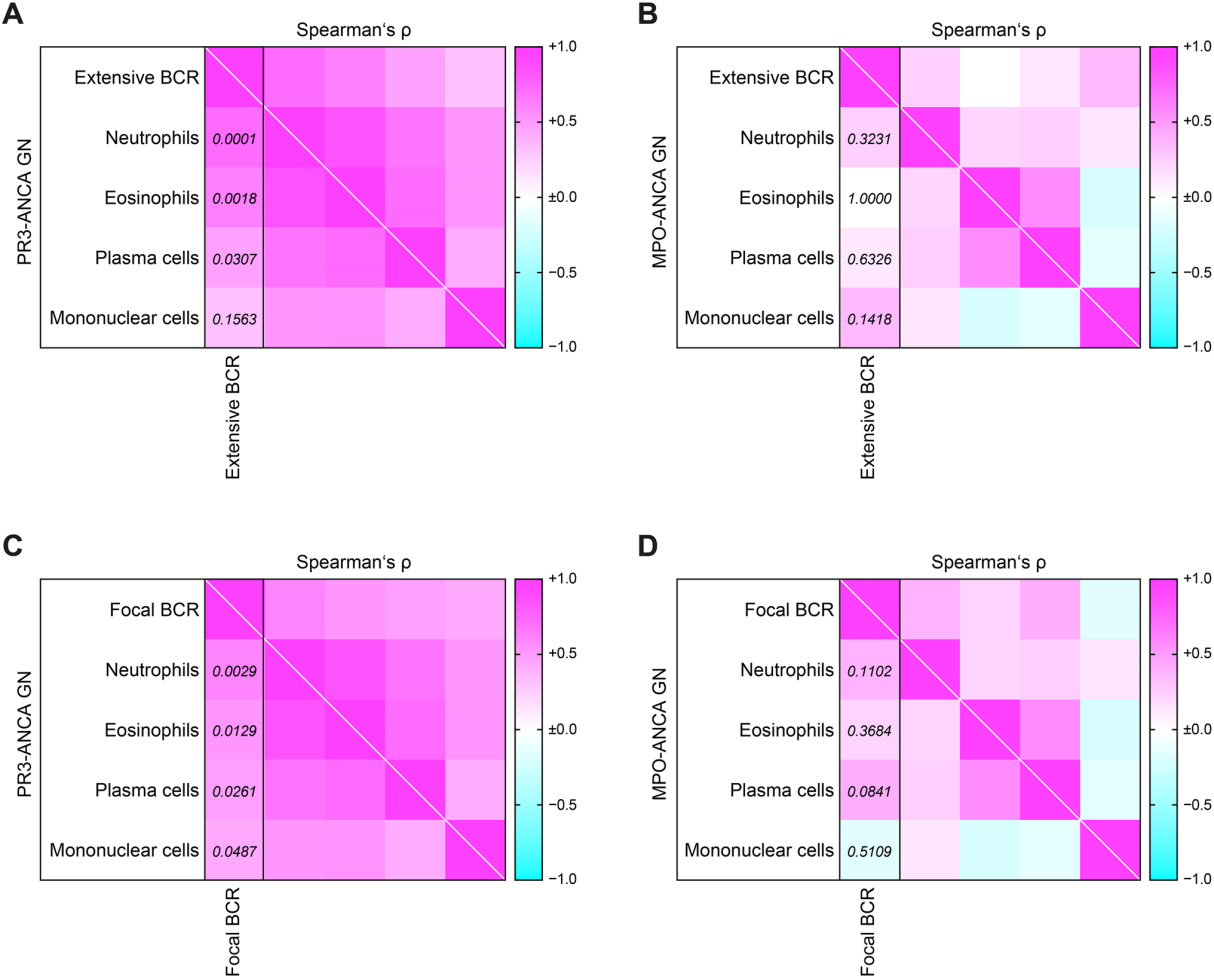
Tubulointerstitial inflammation containing neutrophils associates with BCRspecifically in PR3-ANCA GN. **A**,** B** Association between the fraction of glomeruli affected by extensive BCR and subtypes of immune cell infiltrates separated for PR3-ANCA and MPO-ANCA GN is shown by a heatmap reflecting mean values of Spearman’s ρ, corresponding values of *p* are shown. **C**,** D** The association between the fraction of glomeruli affected by focal BCR and subtypes of immune cell infiltrates separated for PR3-ANCA and MPO-ANCA GN is shown by a heatmap reflecting mean values of Spearman’s ρ, corresponding values of *p* are shown. *ANCA* anti-neutrophil cytoplasmic antibodies, *BCR* Bowman’s capsule rupture, *GN* glomerulonephritis, *MPO* myeloperoxidase, *PR3* proteinase 3

Tubulointerstitial inflammation in ANCA GN contained neutrophils, eosinophils, plasma and mononuclear cells (macrophages and lymphocytes, Fig. 1A). Extensive Bowman’s capsule rupture was associated with tubulointerstitial inflammation containing infiltrates of neutrophils, eosinophils and plasma cells (Fig. 1B, C). In contrast, infiltration of mononuclear cells including macrophages and lymphocytes did not show an association with Bowman’s capsule rupture in ANCA GN (Fig. 1C). A similar association was observed for the presence of focal Bowman’s capsule rupture, correlating with tubulointerstitial inflammation containing neutrophils, eosinophils and plasma cells (Fig. 1D). Multiple logistic regression confirmed that extensive Bowman’s capsule rupture was associated with tubulointerstitial inflammation containing neutrophils (*p* < 0.01, Table 1), and that focal Bowman’s capsule rupture was associated with neutrophil (*p* < 0.01) and plasma cell infiltrates (*p* < 0.05, Table 2). To elucidate the contribution of immune cell infiltration to Bowman’s capsule rupture in distinct ANCA subtypes, we then analyzed PR3-ANCA and MPO-ANCA GN separately. Interestingly, an association between tubulointerstitial inflammation containing neutrophils and Bowman’s capsule rupture was specifically observed in PR3-ANCA GN (Fig. 2A). In contrast, no association between subtypes of immune cell infiltrates and Bowman’s capsule rupture was observed in MPO-ANCA GN (Fig. 2B).

**Table 1:** Multiple regression analysis for immune cell infiltrates associated with extensive BCR in ANCA GN

Immune cell subtype	ß	SE	p value
Neutrophils—%	0.4941	1.4642	**0.0056**
Eosinophils—%	0.2529	2.0512	0.2440
Plasma cells—%	− 0.1531	0.5061	0.4499
Mononucleated cells—%	− 0.1075	0.2692	0.4866

*ANCA* anti-neutrophil cytoplasmic antibodies, *BCR* Bowman’s capsule rupture, *GN* glomerulonephritis, *SE* standard error

Bold indicates statistically significant value

**Table 2:** Multiple regression analysis for immune cell infiltrates associated with focal BCR in ANCA GN

Immune cell subtype	ß	SE	p value
Neutrophils—%	0.4921	3.3055	**0.0056**
Eosinophils—%	− 0.2345	4.6306	0.2779
Plasma cells—%	0.4146	1.1425	**0.0451**
Mononucleated cells—%	− 0.1441	0.6077	0.3508

*ANCA* anti-neutrophil cytoplasmic antibodies, *BCR* Bowman’s capsule rupture, *GN* glomerulonephritis, *SE* standard error

Bold indicates statistically significant value

## Discussion

The performance of the established histopathological subtyping proposed by Berden et al. and Brix et al. has improved with the implementation of Bowman’s capsule rupture to the classification systems [24]. The additional predictive value of Bowman’s capsule rupture is linked to its marker of irreversible nephron damage, as a consequence of segmental glomerulosclerosis caused by Bowman’s capsule rupture.

Moreover, an increased of glomeruli showing Bowman’s capsule rupture in ANCA GN was associated with tubulointerstitial inflammation, further stressing the predictive value of interstitial inflammation in the decline of kidney function in ANCA GN [4, 10, 19].

In addition, we recently characterized intrarenal subtypes of immune cell infiltrates in MPO-ANCA versus PR3-ANCA GN, associated with distinct glomerular and tubulointerstitial lesions [23]. Based on these previous observations, we here aimed to analyze the association between Bowman’s capsule rupture and distinct immune cell infiltrates in ANCA GN. To our knowledge, this is the first report linking Bowman’s capsule rupture directly to tubulointerstitial inflammation by characterizing immune cell subtypes.

We observed that extensive Bowman’s capsule rupture correlated with tubulointerstitial inflammation containing neutrophils, and that focal Bowman’s capsule rupture correlated with neutrophil and plasma cell infiltration. This association was specifically observed in PR3-ANCA GN [25].

At disease onset, neutrophils are activated by pathogenic ANCAs, causing a release of inflammatory cytokines, reactive oxygen species, and lytic enzymes, thus resulting in an excessive formation of neutrophil extracellular traps (NETs) [26]. Intrarenal infiltrates of primed neutrophils are activated by MPO-ANCA and PR3-ANCA, leading to degranulation with release of cytoplasmic granules into the glomerular and interstitial space. As a result, reactive oxygen radicals (RORs) accumulate and cause vascular damage [26]. In addition, stimulation of neutrophils causes the release of factors that activate the alternative complement system, aggravating vascular damage [26]. Small vessel leakage of serum proteins and the formation of fibrin give rise to fibrinoid necrosis. Besides neutrophils, plasma cell infiltration is also commonly observed in ANCA GN and has been associated with tubulointerstitial inflammation and IgG4 positivity [27]. While neutrophil and plasma cell infiltrates predominate the early phase of inflammation in AAV, necrotizing lesions evolve into granuloma rich in monocyte/macrophages, and this is accompanied by infiltration of lymphocytes, such as T cells [28, 29]. Our observation that an association between neutrophil infiltration and Bowman’s capsule rupture was specifically seen in PR3-ANCA GN suggests distinct pathogenetic mechanisms in ANCA subtypes, requiring further investigation.

The main limitations of our study are its retrospective design in a single center and a selection bias towards more severe cases of ANCA GN with limited information on kidney function before the kidney biopsy.

Nevertheless, Bowman’s capsule rupture has independently been associated with poor renal outcome in ANCA GN [24].

This underscores the need for further studies with regard to specific immune cell infiltrates as a potential therapeutic target in distinct subtypes of severe ANCA GN.

## Data Availability

Deidentified data are available on reasonable request from the corresponding author.
